# Evaluation of Saccharin and Resveratrol as Extrinsic Markers of Small-Quantity Lipid-Based Nutrient Supplement Consumption in Healthy Women

**DOI:** 10.1093/cdn/nzab089

**Published:** 2021-07-06

**Authors:** Sarah J Zyba, Valerie Weinborn, Charles D Arnold, Arlie L Lehmkuhler, Fanny B Morel, Mamane Zeilani, Alyson E Mitchell, Marjorie J Haskell

**Affiliations:** Institute for Global Nutrition, Department of Nutrition, University of California, Davis, Davis, CA, USA; Department of Food Science and Technology, University of California, Davis, Davis, CA, USA; Institute for Global Nutrition, Department of Nutrition, University of California, Davis, Davis, CA, USA; Department of Food Science and Technology, University of California, Davis, Davis, CA, USA; Nutriset S.A.S., Malaunay, France; Nutriset S.A.S., Malaunay, France; Department of Food Science and Technology, University of California, Davis, Davis, CA, USA; Institute for Global Nutrition, Department of Nutrition, University of California, Davis, Davis, CA, USA

**Keywords:** adherence marker, resveratrol, saccharin, small-quantity lipid-based nutrient supplement, *trans*-resveratrol

## Abstract

**Background:**

Dietary supplements, like small-quantity lipid-based nutrient supplements (SQ-LNS), are used in intervention programs to prevent undernutrition among women and young children in low-income countries. An objective marker is needed to track consumption of supplements to evaluate the effectiveness of these programs.

**Objective:**

The aim of this study was to evaluate saccharin and resveratrol as potential adherence markers for tracking recent consumption of a single serving of SQ-LNS in women.

**Methods:**

Forty-seven healthy nonpregnant women 18–45 y of age were assigned to consume a single dose of SQ-LNS (20 g) containing either 10 mg sodium saccharin or 5 mg *trans*-resveratrol, under supervision. On the day before and for 2 d following SQ-LNS consumption, urine samples were collected each day for 24 h as 3 consecutive 4-h collections and one 12-h overnight collection. Urinary concentrations of saccharin and *trans*-resveratrol-3-O-sulfate, a resveratrol metabolite, were measured by ultra-high-performance liquid chromatography interfaced to a mass spectrometer with electrospray ionization [UHPLC-(ESI-)MS/MS]. Urinary concentrations (μmol/L urine) of saccharin and *trans*-resveratrol-3-O-sulfate were plotted against time, and receiver operating characteristic (ROC) curves were used to determine the discriminative capacity of each compound, at each post-consumption time point compared with baseline, to detect recent consumption of SQ-LNS. Cutoff values to differentiate supplement consumption from nonconsumption of each marker were developed using the closest-to-(0,1)-corner cut-point approach.

**Results:**

Forty-five participants were included in the analysis. Urinary concentrations of saccharin and *trans*-resveratrol-3-O-sulfate increased within 4 h of SQ-LNS consumption. Urinary concentration cutoff values for saccharin (13.4 µmol/L) and *trans*-resveratrol-3-O-sulfate (0.7 µmol/L) allowed for 78% and 89% sensitivity, respectively, and 100% specificity in detecting consumption of SQ-LNS within the first 12 h after consumption.

**Conclusions:**

Urinary concentrations of saccharin and *trans*-resveratrol-3-O-sulfate reflect consumption of SQ-LNS containing those compounds during the first 12 h post-consumption with high sensitivity and specificity in healthy women and may be useful objective adherence markers for tracking consumption of SQ-LNS.

## Introduction

Nutrition programs that provide food or nutrient supplements to populations at risk of deficiencies are an important strategy for preventing maternal and childhood undernutrition in low-income countries (LICs). For example, small-quantity lipid-based nutrient supplements (SQ-LNS) are provided to pregnant and lactating women and young children in LICs to prevent undernutrition (1–4). SQ-LNS are based on peanut paste, and provide energy, protein, and essential fatty acids, and are enriched with vitamins and minerals to meet the needs of specific target populations. SQ-LNS can be consumed alone or mixed with foods prepared in the household, such as porridge. Evaluation of the health impacts of nutrition programs or intervention trials, such as those providing SQ-LNS, requires information on whether the food or nutrient supplement was actually consumed by the intended recipients. If no effect is observed on nutrition or health outcomes, it is important to assess whether the lack of effect may be due to insufficient consumption of the supplement by the target recipients. Currently, adherence (i.e., the consumption of a nutrient supplement according to an intervention protocol) is assessed in community-based nutrition trials through self-report, product disappearance rate, and, when possible, by direct observation (1, 2). While these methods can be useful, reported use and the product disappearance rate may not be reliable, and direct observation is not feasible in large, community-based trials. Components of the intervention products themselves, such as of vitamins, minerals, or other bioactive compounds, may be useful as adherence markers in some contexts if consumption of the product results in an increase in plasma and/or urine concentrations of these components. However, these are not ideal adherence markers because they may be found in other foods or food products in the diet, and it may be difficult to attribute a response solely to the intervention product. Moreover, an increase in plasma or urine concentrations of a component of the intervention product may indicate adherence, but lack of response does not necessarily indicate nonadherence. For example, a program participant may consume a supplement as instructed, but the amount of the component in the supplement may not be sufficient to detect a significant biological response or there may be other participant-related factors such as infection or inflammation that impact the response. Objective methods using an extrinsic marker for assessing adherence to food and nutrient supplements are limited. Absorbable markers, such as lithium (3) and para-amino benzoic acid (4), have been used in previous clinical studies with adults; however, there is concern about the safety of their long-term daily administration, which would be necessary in nutrition supplementation studies that frequently last months to a year, and about the safety of the use of these markers in children.

It may be possible to develop an objective method for assessing adherence qualitatively by identifying an extrinsic marker that is safe for long-term, daily administration and that can be tracked in spot urine samples. This approach is more practical for use in young children in large-scale community-based trials in LICs, and an objective measure is likely to be more reliable than reported adherence or product disappearance rates.

Two compounds that may work well as markers of adherence are saccharin and resveratrol. Saccharin is an FDA-approved nonnutritive sweetener that is not metabolized by the body (5–8). It is often used to replace sucrose in some low-/noncalorie food items and as an alternative to table sugar packets. Existing pharmacokinetic data suggest that urinary concentrations of saccharin reflect recent intake (9, 10). Resveratrol is a compound found in red grapes and is considered Generally Recognized As Safe by the US FDA and approved for use as a supplement by the European Food Safety Authority (11). Resveratrol is rapidly metabolized and excreted in urine, and urinary concentrations of resveratrol metabolites have been used in epidemiological studies as a marker of red wine intake (12–14). Both compounds are not likely to be found in high amounts in the diets of women and young children in LICs, although this would need to be confirmed prior to considering either compound as an adherence marker.

Although SQ-LNS are typically consumed by pregnant and lactating women or young children in LICs, as a first step for developing an adherence marker we conducted a proof-of-concept pilot study in healthy nonpregnant women in the United States. The primary objective of the study was to evaluate saccharin and resveratrol as potential markers for tracking consumption of a single serving of SQ-LNS and to identify the optimal time point(s) for measuring urinary concentrations of saccharin and resveratrol metabolites.

## Methods

### Participants

Forty-seven healthy, nonpregnant, nonlactating women 18–45 y of age with a BMI (in kg/m^2^) between 20 and 25 who reported being free from chronic diseases, did not smoke, consume excessive alcohol, or use any medications affecting gastrointestinal mobility; had no peanut, cow milk, soy, or artificial sweetener allergies; and were willing to refrain from consuming dietary sources of the marker compounds during the study were recruited from Davis, California, to participate in this study. Potential participants were screened for eligibility, and written informed consent was obtained from eligible women who chose to participate in the study before beginning any procedures. The study was conducted at the Ragle Human Nutrition Center on the University of California (UC), Davis, campus and was approved by the UC Davis Institutional Review Board.

### Study procedures

**FIGURE 1:**
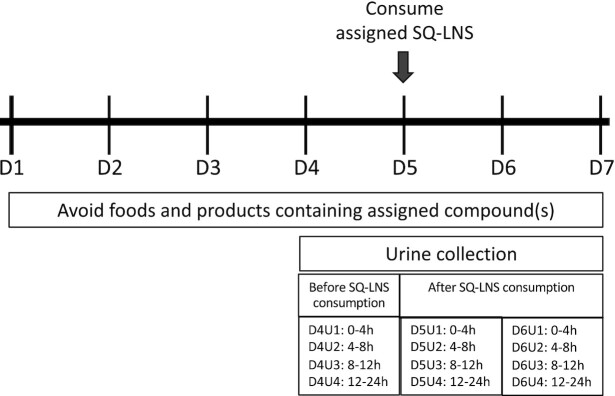
Schematic diagram of the study protocol, by study day 1–6, for a study in which 47 healthy women consumed a single 20-g dose of SQ-LNS containing either 10 mg sodium saccharin or 5 mg resveratrol. Urine samples collected over 24 h, collected in 4-h (U1–3) or 12-h (U4) periods, were collected the day before SQ-LNS consumption (D4) and for 2 d after (D5 and D6). SQ-LNS were consumed with a small portion of rice porridge the morning of D5. SQ-LNS, small-quantity lipid-based nutrient supplement(s).

The study was conducted between April and July 2018. Participants were assigned to receive a single dose of either SQ-LNS (20 g) containing 10 mg sodium saccharin (SQ-LNS/S) or 5 mg of *trans*-resveratrol (SQ-LNS/R). The study was not masked; participants and researchers were aware of the SQ-LNS product assignments. The first 23 participants received SQ-LNS/R and the remaining 23 participants received SQ-LNS/S. The SQ-LNS was provided by Nutriset S.A.S. (Malaunay, France) and packaged in individual sachets. The SQ-LNS used in this study was designed for pregnant and lactating women and contained peanut paste, milk powder, and vegetable oils, and is enriched with vitamins and minerals; each 20-g sachet provides 118 calories. The nutrient content of SQ-LNS is described in **Supplemental Table 1**. Food-grade sodium saccharin (Sodium Saccharin 15% hydrated; BENEO GmbH) and food-grade *trans*-resveratrol (resVida; DSM Nutritional Products) were added to the SQ-LNS during production. Participants were provided with a list of foods and products that contain their assigned target compound and were asked to refrain from consuming any foods or products on the list on study days 1–3 (washout) and for the duration of the study (study days 4–6) (**Figure 1**).

### Baseline urine collection

On study day 4 (D4), participants came to the Ragle Human Nutrition Center at 08:00 and were interviewed briefly to obtain information on any consumption of their assigned target compound or any symptoms of illness in the 3-d washout period. Thereafter, participants were instructed to collect urine samples during the next 24-h period. Urine was collected in 3 consecutive 4-h time periods [∼08:00–12:00 (U1); 12:00–16:00 (U2); 16:00–20:00 (U3)] and in one 12-h (overnight) period [20:00–08:00 (U4)]. Participants were given separate urine collection containers for each collection period in a small cooler with ice packs. All urine produced during a collection period was pooled into 1 container. Participants returned their morning (U1) and afternoon (U2) urine containers to study staff at the Ragle Human Nutrition Center at ∼16:00 and were given 2 more containers for the evening (16:00–20:00, U3) and overnight (20:00–08:00, U4) collection periods in a small cooler with fresh ice packs. Urine samples collected during the evening and overnight were returned to Ragle the following morning by ∼08:00. Urine was processed by the study staff and frozen at –80°C until analysis.

### Administration of SQ-LNS and urine collection

On day 5 (D5), during their morning visit, participants were interviewed briefly to obtain information on any consumption of their assigned target compound and symptoms of illness in the previous 24 h. They were then given 1 packet of SQ-LNS (20 g) containing their assigned compound mixed with 0.25 serving (11 g) of rice porridge (Cream of Rice; Nabisco) prepared with water per packaging instructions and 2 packets of sugar (0.1 oz pure cane non-GMO granulated sugar; C&H) added. Participants were instructed to consume the entire portion and were monitored for 30 min after consumption to ensure that they did not eat or drink anything but water. Thereafter, participants were asked to collect their urine for the next 24-h period using the procedures described above. On the morning of study day 6 (D6), participants were interviewed to obtain information on any consumption of their assigned target compound or symptoms of illness in the past 24 h and were asked to collect their final 24-h urine sample using the same procedures.

### Stability testing of markers in SQ-LNS

To evaluate the shelf-life of the marker compounds in SQ-LNS at temperatures typical of tropical countries, samples of SQ-LNS/S (containing 10 mg sodium saccharin) and SQ-LNS/R (containing 5 mg *trans*-resveratrol) were stored at 30°C and 40°C for 25 mo post-production at the Nutriset production facility. Samples of each SQ-LNS product were shipped to UC Davis for analysis at times 0, 3, 6, 12, and 25 mo post-production.

### Urine processing

After collecting urine containers from participants, the containers were stored at 4°C until processed; all urine was processed within ∼2 h of receipt. Urine volume was measured and recorded for each container. Thirty milliliters of urine was then transferred to a 50-mL conical tube containing 60 mg boric acid (99% boric acid; Sigma) and mixed thoroughly to dissolve the boric acid. Three 2-mL samples of the boric acid–preserved urine was then transferred into cryovials and stored at −80°C until analysis. Remaining urine was discarded. To protect resveratrol from UV-light degradation, all urine processing was completed in a room with dimmed lights, and the cryovials were wrapped in aluminum foil to protect the samples until analysis.

### Urinary creatinine analysis

Creatinine was measured using a colorimetric assay (Creatinine Assay Kit; Cellbiolabs) following the manufacturer's protocol and analyzed on a BioTek Synergy H1 plate reader. All samples from a single participant were analyzed at the same time, in duplicate.

### Analysis of saccharin, *trans*-resveratrol, and resveratrol metabolites in urine

To measure urinary concentrations of the adherence marker compounds, urine samples were thawed at room temperature (25°C) for 2 h and diluted 10 (*trans*-resveratrol) or 50 (saccharin) times in methanol:water (1:1). Urine samples containing saccharin had 100 ng/mL of saccharin-d4 (CDN Isotopes) added, and were vortexed for 5 s. Samples were transferred to an autosampler vial for analysis by Agilent 1290 Infinity Ultra-HPLC (UHPLC) interfaced to a 6460 triple-quadrupole mass spectrometer (MS/MS) with electrospray ionization (ESI) via Jet Stream technology (Agilent Technologies) (15). Resveratrol metabolites were expressed as resveratrol equivalents. Recoveries for this method have been shown to be >80% at the range of concentrations used in this study.

### Analysis of saccharin and *trans*-resveratrol in SQ-LNS

SQ-LNS sachets (*n* = 3 sachets per time point at both storage temperatures) were thawed at room temperature and homogenized by hand for 15 s each. A sample of 1 g (±0.1 g) was weighed from each sachet in triplicate and transferred to UV-light–protected 50-mL tubes. Ten milliliters of water and 10 mL of acetonitrile were added, and the mixture was vortexed for 30 s after each addition. QUECHERS salts (Agilent Technologies), containing 4 g of magnesium sulfate (MgSO_4_) and 1.5 g of sodium acetate (NaOAc), were added and the mixture was vortexed for 1 min. Samples were centrifuged at 4000 × *g* for 10 min at room temperature. From the top layer (acetonitrile), 25 µL was transferred to an autosampler vial containing 875 µL methanol:water (1:1) and 100 µL of the corresponding surrogate [*trans*-resveratrol d4 (1 µg/mL) or saccharin d4 (1 µg/mL)] for analysis by UHPLC-(ESI)-/MS/MS (15). This method was validated showing recoveries of 100% for saccharin and 92% for resveratrol.

### Urinary elimination of compounds

The mean urinary concentrations of saccharin and resveratrol metabolites were plotted by urine collection period (D5U1–D6U4) to assess the time course of urinary elimination of the compounds during 48 h after consumption of the SQ-LNS.

### Cumulative excretion of the compounds

The total amount of each compound excreted in urine during the 48-h collection period after consumption of the SQ-LNS was calculated for each participant and expressed as the percentage of the administered oral dose. This was done by multiplying the concentration of each compound in urine by urine volume for each collection period, summing the amounts for all collection periods post–SQ-LNS consumption, and dividing the total amount excreted by the dose administered.

### Sample size and statistical analysis

The sample size of 20 participants per SQ-LNS product was based on the number of participants enrolled in a study designed to validate urinary resveratrol concentration as a biomarker of wine intake (16). The sample size was increased by 15% to account for attrition (23 participants for each SQ-LNS product; total of 46 participants).

The pre-declared primary endpoints of this study were urinary concentrations of saccharin and *trans*-resveratrol-3-O-sulfate and these did not change during the study. Descriptive statistics were calculated for all variables and marker compound variables were log-transformed for analyses that assumed normal distributions. Results were back-transformed to concentration units for presentation. Change in concentration of the markers in SQ-LNS over time was determined using 1-factor ANOVA.

### Estimation of discriminating power

To determine which urine collection period had the best discriminating power to detect consumption of SQ-LNS, the urinary concentrations of the compounds at each post-consumption collection period (D5U1–D6U4) were compared with urinary concentrations that were measured pre-consumption (D4U1–D4U4). Using logistic regression, receiver operating characteristic (ROC) curves were developed for each comparison of post-consumption collection period versus pre-consumption collection period. The optimal post-consumption collection period was determined by comparing the mean (95% CI) estimates of area under the receiver operating characteristic (AUROC) curve among collection periods. To establish an exploratory value for categorizing participants as consumers or nonconsumers we applied the closest-to-(0,1)-corner cut-point approach (17) to the D5U1–U3 time points (0–12 h after consumption of SQ-LNS containing the markers). The closest-to-(0,1)-corner cut-point approach defines the optimal cut-point as the point in the ROC curve closest to perfect sensitivity and perfect specificity. These time points were selected to detect consumption of the supplement within the past 12 h.

### Pilot study in children

A very small proof-of-concept study was conducted in children 12–24 mo of age to determine whether urinary concentrations of saccharin and *trans*-resveratrol-3-O-sulfate would reflect recent intake of SQ-LNS in response to consumption of a single dose of SQ-LNS (10 g) containing both 5 mg of saccharin and 5 mg *trans*-resveratrol. Four apparently healthy children in Davis, California, were enrolled in the study. The study protocol was approved by the Institutional Review Board at UC Davis, and written informed consent was obtained from at least 1 parent/guardian of each child. The study was conducted in participants’ homes due to COVID-19 (coronavirus disease 2019) restrictions (**Supplemental Methods**). Briefly, a baseline spot urine sample (∼10 mL) was collected before SQ-LNS consumption and then spot urine samples were collected from all urinary voids for the following 6 h. A final spot urine sample was collected the following morning ∼24 h after SQ-LNS consumption.

## Results

### Participant characteristics

**FIGURE 2:**
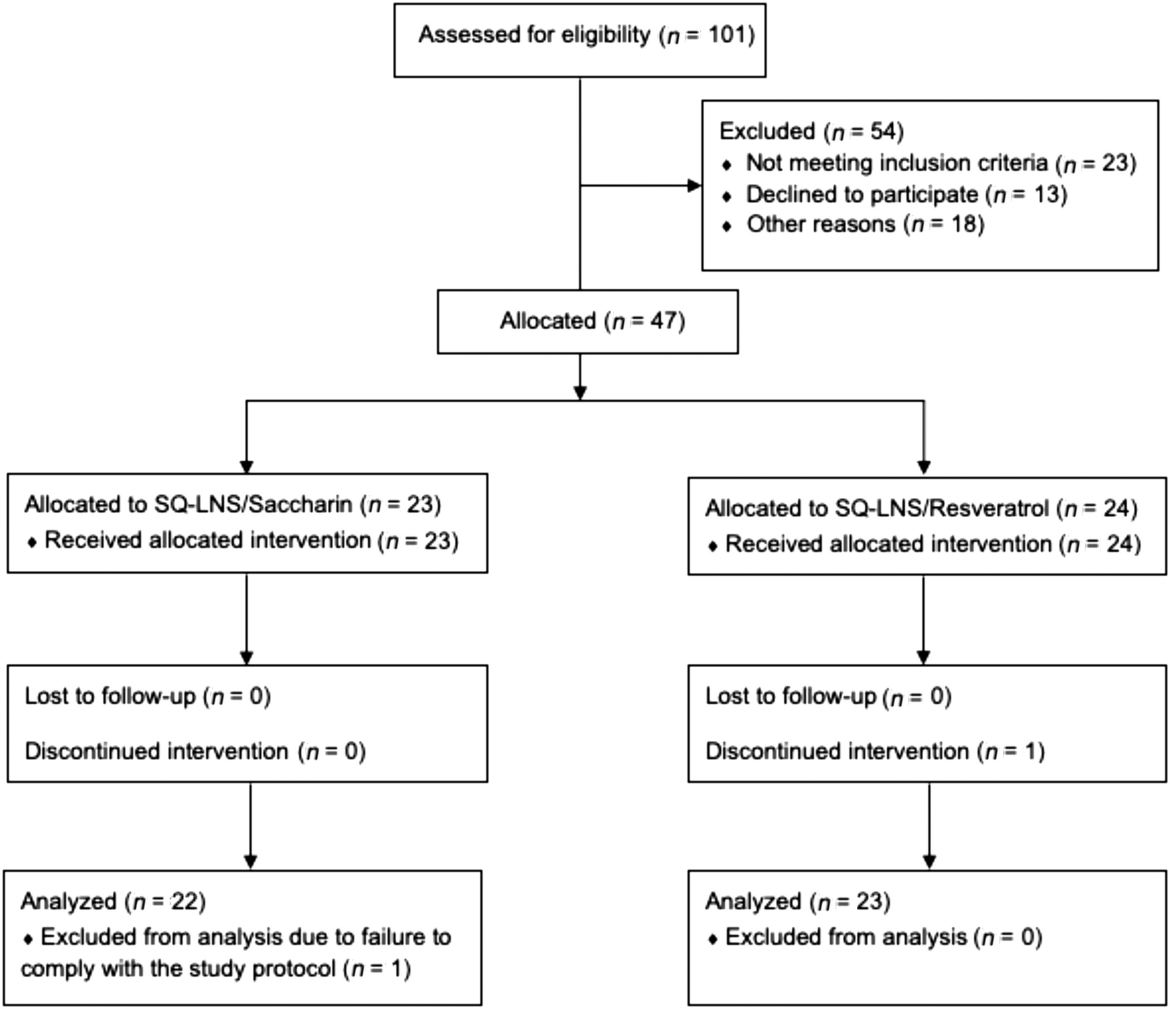
Flowchart of participant progression through a study in which 47 healthy women consumed a single 20-g dose of SQ-LNS containing either 10 mg sodium saccharin or 5 mg resveratrol. SQ-LNS, small-quantity lipid-based nutrient supplement(s).

Of the 101 women assessed for eligibility, 47 were enrolled and 46 participants completed the study; 1 individual in the SQ-LNS/S group withdrew consent prior to collection of post–SQ-LNS consumption urine samples and is not included in the analyses. An additional participant from the SQ-LNS/S group was excluded from the analyses due to failure to comply with the study protocol (**Figure 2**). The mean participant age and BMI are reported in **Table 1**.

**TABLE 1:** Characteristics of study participants who were assigned to consume a single serving of SQ-LNS with 10 mg saccharin or 5 mg *trans*-resveratrol^1^

	SQ-LNS/S (*n* = 23)	SQ-LNS/R (*n* = 24)
Age, y	24 ± 5	25 ± 5
Height, cm	166.3 ± 5.5	163.1 ± 6.1
Weight, kg	61.5 ± 5.5	60.3 ± 7.1
BMI, kg/m^2^	22 ± 2	23 ± 2

1 Values are means ± SDs. All participants are female. SQ-LNS, small-quantity lipid-based nutrient supplement; SQ-LNS/R, small-quantity lipid-based nutrient supplement containing *trans-*resveratrol; SQ-LNS/S, small-quantity lipid-based nutrient supplement containing sodium saccharin.

### Marker compound content and stability in SQ-LNS

At the time of production, SQ-LNS/S (20 g) contained 9.4 ± 0.5 mg sodium saccharin (8.3 mg saccharin), on average, and SQ-LNS/R (20 g) contained 4.6 ± 0.1 mg *trans*-resveratrol, on average, which was close to the target amounts of 10 mg sodium saccharin and 5 mg *trans*-resveratrol (**Supplemental Table 2**). Sodium saccharin in SQ-LNS was stable out to 25 or 12 mo post-production when stored at 30°C or 40°C, respectively. However, there was some variation over time of *trans*-resveratrol content in SQ-LNS at 30°C (*P* = 0.004) and a slight decrease over time at 40°C (*P* < 0.001), with ∼91% retention of the compound at 3 mo after production and 83% retention by 25 mo after production.

### Urinary excretion of saccharin

**FIGURE 3:**
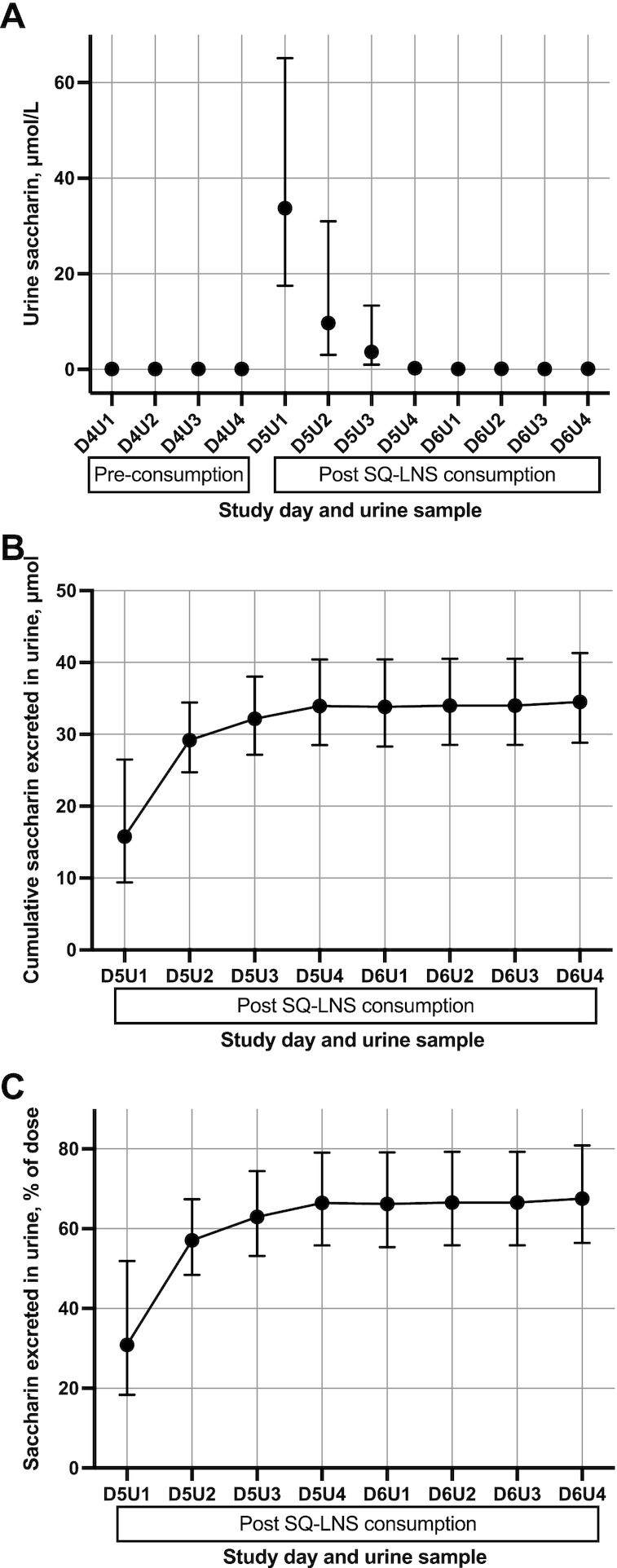
Urinary concentrations and cumulative excretion of saccharin for 22 women who consumed 9.4 mg sodium saccharin in SQ-LNS. Values are geometric means (95% CIs). (A) Urinary saccharin concentrations (μmol/L urine) pre-consumption (D4U1–D4U4), 0–24 h after consumption (D5U1–D5U4), and 24–48 h after consumption (D6U1–D6U4). (B) Cumulative excretion of saccharin in urine (µmol) 0–48 h after consumption (D5U1–D6U4). (C) Cumulative percentage of the 9.4-mg sodium saccharin dose excreted in urine 0–48 h after consumption. SQ-LNS, small-quantity lipid-based nutrient supplement(s).

Urinary saccharin and *trans*-resveratrol both had a geometric mean (95% CI) concentration of 0.1 (0.1, 0.1) μmol/L before SQ-LNS consumption (D4U1–D4U4). Urinary saccharin concentrations peaked between 0 and 4 h after consumption of SQ-LNS/S at 33.7 (17.5, 65.0) μmol/L (D5U1). Thereafter, mean urinary saccharin concentrations declined and were close to baseline concentrations by 24 h after consumption (D5U4) (**Figure 3**A). The mean (95% CI) cumulative urinary excretion of saccharin was 34.5 μmol (6.3 mg) (28.8, 41.3 μmol) at 48 h after consumption of SQ-LNS/S, and 98.3% of this was excreted within the first 24 h [33.9 μmol (6.2 mg) (28.5, 40.4)] (Figure 3B). The mean (95% CI) percentage of saccharin dose recovered in urine was 67.5% (56.4%, 80.8%) (Figure 3C).

### Urinary excretion of resveratrol metabolites

**FIGURE 4:**
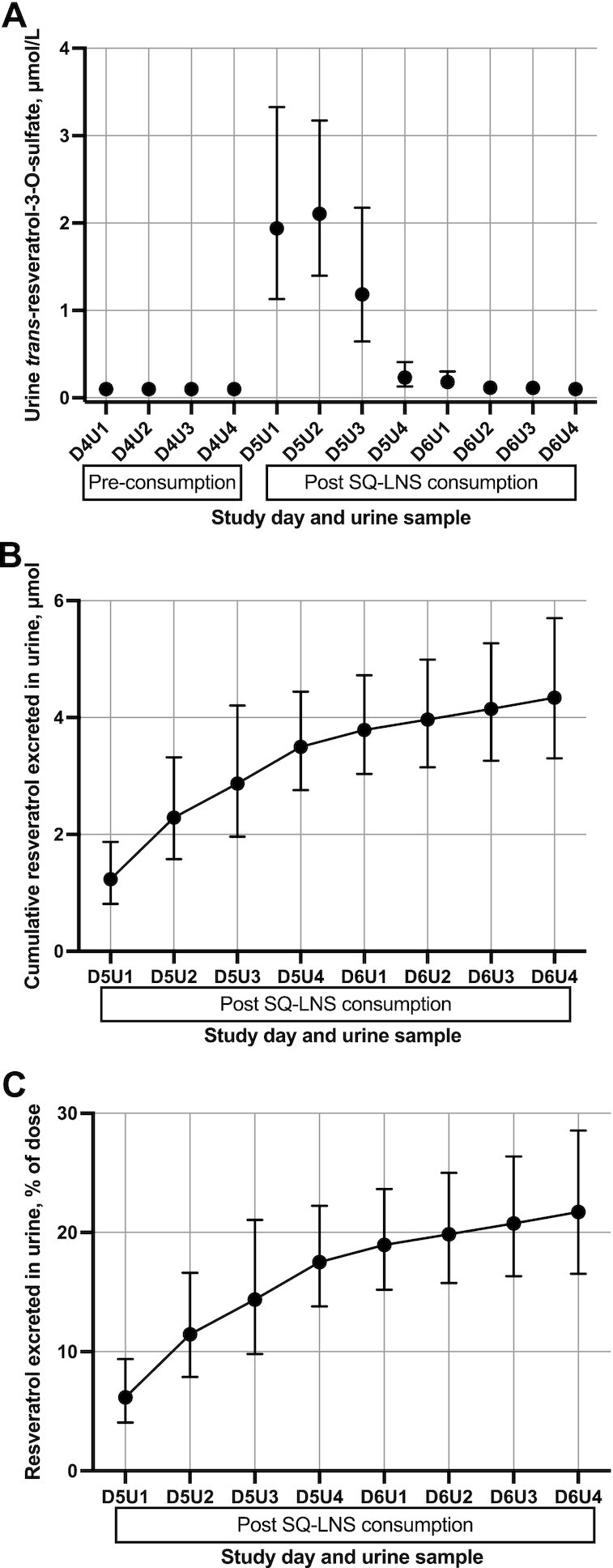
Urinary concentrations and excretion of resveratrol metabolites for 23 women who consumed 4.6 mg of *trans*-resveratrol in SQ-LNS. Values are geometric means (95% CIs). (A) Urinary *trans*-resveratrol-3-O-sulfate concentrations (μmol/L urine) pre-consumption (D4U1–D4U4), 0–24 h after consumption (D5U1–D5U4), and 24–48 h after consumption (D6U1–D6U4). (B) Cumulative excretion of measured resveratrol metabolites in urine (µmol) 0–48 h after consumption (D5U1–D6U4). (C) Cumulative percentage of the 4.6-mg *trans*-resveratrol dose excreted in urine 0–48 h after consumption. SQ-LNS, small-quantity lipid-based nutrient supplement(s).

In preliminary analyses, we found that *trans-*resveratrol-3-O-sulfate constituted 69% of the total measured resveratrol metabolites in the urine (15). For this reason, *trans*-resveratrol-3-O-sulfate was chosen as the optimal metabolite for tracking consumption of SQ-LNS. *trans*-Resveratrol-3-O-sulfate excretion was highest during the first 8 h after consumption of SQ-LNS/R; the mean (95% CI) concentration was 1.9 (1.1, 3.3) μmol/L from 0 to 4 h (D5U1) and 2.1 (1.4, 3.2) μmol/L from 4 to 8 h (D5U2) (**Figure 4**A). Urinary *trans*-resveratrol-3-O-sulfate concentrations were close to baseline concentrations by 24 h (D5U4) after consuming the dose. To estimate the percentage of resveratrol dose recovered in urine, all measured urinary resveratrol metabolites were combined; this included urinary concentrations of *trans*-resveratrol-3-O-glucuronide, *trans*-resveratrol-4-O-glucuronide, and *trans*-resveratrol-3-O-sulfate. The mean (95% CI) cumulative excretion of resveratrol metabolites was 4.3 μmol (0.99 mg) (3.3, 5.7 μmol) at 48 h after consumption (Figure 4B) and the mean (95% CI) percentage of resveratrol dose recovered in urine was 21.7% (16.5%, 28.5%) (Figure 4C).

### Discriminating power for detecting consumption of SQ-LNS

**FIGURE 5:**
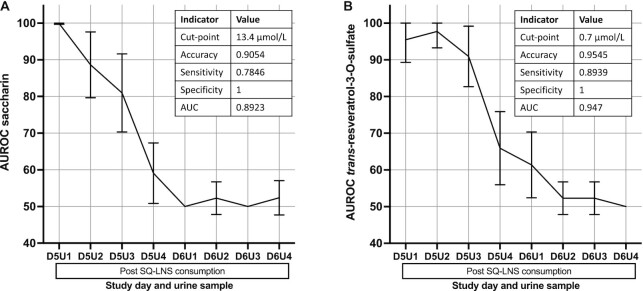
Discrimination power by time point and optimal urinary concentration cut-point to discriminate between consumption and nonconsumption up to 12 h after SQ-LNS consumption for saccharin (*n* = 22 women) (A) and *trans*-resveratrol-3-O-sulfate (*n* = 23 women) (B). Urine samples were collected over 4-h (U1–U3) or 12-h (U4) periods the day before SQ-LNS consumption (D4) and for 2 d following SQ-LNS consumption (D5–D6). SQ-LNS were consumed the morning of D5. ROC curves were developed for each comparison of post-consumption collection period vs. baseline (pooled D4U1–D4U4). The closest-to-(0,1)-corner cut-point approach was used with the D5U1–D5U3 time points to determine a cut-point to categorize participants as having consumed or not consumed the supplement within the last 12 h. AUROC, area under the receiver operating characteristic curve; ROC, receiver operating characteristic; SQ-LNS, small-quantity lipid-based nutrient supplement(s).

The AUROC for both saccharin and *trans*-resveratrol-3-O-sulfate was >89% for all samples collected within the first 12 h after consumption (D5U1–D5U3) (**Figure 5**). Therefore, a cutoff point for discriminating between consumption and nonconsumption was based on pooled data on urinary concentrations of the compounds from urine samples D5U1–D5U3. A cutoff of 13.4 μmol/L urine for saccharin allowed for ∼78% sensitivity and 100% specificity (Figure 5A). A cutoff of 0.7 μmol/L urine for *trans*-resveratrol-3-O-sulfate allowed for ∼89% sensitivity and 100% specificity (Figure 5B).

Mean urine concentrations of saccharin and *trans*-resveratrol-3-O-sulfate were also expressed per gram of creatinine (**Supplemental Table 3**). However, there were no differences in the sensitivity and specificity of the cutoff values based on per-unit volume compared with per-gram creatinine and very few participant samples were classified as indicating “consumption” versus “nonconsumption” of the compounds differently based on the cutoff values when expressed as per-unit urine volume or per-gram creatinine (**Table 2**). The percentage disagreement was calculated based on the proportion of participants who were classified differently depending on the expression of urinary concentrations.

**TABLE 2:** Comparison of participant sample classifications using urinary concentrations of saccharin and *trans*-resveratrol-3-O-sulfate expressed per liter of urine volume or per gram of urinary creatinine^1^

	Saccharin, μmol/g creatinine		Overall disagreement^2^
	Nonconsumption	Consumption	
Saccharin in μmol/L urine			
Nonconsumption	199	2	5/256 (2%)
Consumption	3	52	
			
	*trans*-Resveratrol-3-O-sulfate in μmol/g creatinine		
*trans*-Resveratrol-3-O-sulfate in μmol/L urine			
Nonconsumption	191	0	1/264 (0.4%)
Consumption	1	72	

1 The cutoff points determined for saccharin were 13.4 μmol/L urine and 17.2 μmol/g creatinine and the cutoff points determined for *trans*-resveratrol-3-O-sulfate were 0.7 μmol/L urine and 0.6 μmol/g creatinine. Participant samples (*n *= 256 urine samples collected from 22 women for saccharin and *n* = 264 from 23 women for *trans*-resveratrol-3-O-sulfate in μmol/L urine) were classified as indicating “consumption” or “nonconsumption” of the markers based on the cutoff points per unit volume and per gram of creatinine for each marker.

2 Percent overall disagreement was calculated based on the proportion of samples for which there was disagreement in their classification.

### Child study

Three of 4 enrolled children completed the study (**Supplemental Table 4**); 1 participant withdrew before consuming the SQ-LNS due to challenges with urine collection. Urinary concentrations of both saccharin and *trans*-resveratrol-3-O-sulfate increased within 1 h of SQ-LNS consumption and remained elevated during the 6-h post-consumption collection period (**Supplemental Figure 1**) for all participants. For 2 out of 3 participants, urinary concentrations decreased to below the level of detection by 24 h after consumption.

## Discussion

We found that consumption of SQ-LNS containing either 9.4 mg sodium saccharin or 4.6 mg *trans*-resveratrol as an adherence marker increased urinary concentrations of saccharin or the resveratrol metabolite *trans*-resveratrol-3-O-sulfate within 4 h of consumption and that by using a cutoff value of 13.4 μmol/L for urinary saccharin, or 0.7 μmol/L for urinary *trans*-resveratrol-3-O-sulfate, it was possible to distinguish between consumption and nonconsumption of SQ-LNS up to 12 h after consumption, with high sensitivity and specificity. These results indicate that both compounds reflect recent intake and could potentially be useful to track consumption of SQ-LNS in nutrition intervention trials.

The absorption and rapid excretion of both saccharin and resveratrol seen in this study indicate urinary concentrations reflect recent intake and is consistent with previous pharmacokinetic studies (5, 10, 18, 19). However, the effect of the food matrix on the absorption and excretion of the compounds needs to be considered. In our study, saccharin and *trans*-resveratrol were consumed as part of a high-lipid food matrix (the SQ-LNS), whereas in pharmacokinetic studies of these compounds, the delivery method was typically in a liquid matrix or as a tablet consumed with water. In several studies with a range of doses, recovery of saccharin from a 24-h urine collection was ∼84–95% of the dose (6, 10); and in at least 4 of those studies, the dose was provided as either a capsule consumed with water (5, 10, 20) or in a water-based solution (10, 21). In our study, the mean percentage of saccharin recovered in urine 24 h after consumption was 66%, which suggests absorption of saccharin may be reduced when consumed in a high-lipid food matrix.

*trans*-Resveratrol absorption tends to be low to moderate (5–26% of the dose) (19, 22–25) across a range of doses (1.2–25 mg) when provided in liquid matrices (water, juice, or wine) or as a tablet, and excretion occurs rapidly (19). In Boocock et al. (19), a concentrated oral dose of 500 mg resveratrol was given to 10 healthy adults and 11.4% of the dose was excreted as *trans*-resveratrol-3-O-sulfate during a 24-h urine collection, with most of this (77%) occurring within the first 4 h. The total percentage excreted was close to our results of 17.5% for *trans*-resveratrol-3-O-sulfate within the first 24 h. However, excretion was not as rapid in our study; only 28% of excretion of *trans*-resveratrol-3-O-sulfate occurred within the first 4 h after consumption. This suggests that total absorption of resveratrol may not be impacted by the food matrix, but that the rate of absorption and excretion may be affected. Other studies showed no difference in absorption when resveratrol was consumed as wine compared with grape or vegetable juice, but total absorption was reduced when resveratrol was consumed in the form of a tablet (22, 23).

In intervention programs, it is common for participants to consume SQ-LNS mixed with porridge. For this reason, our study was designed to assess whether the amounts of the markers added to SQ-LNS would be sufficient for tracking consumption in urine when SQ-LNS was consumed with porridge. Although the markers performed well in healthy women and children, they will need to be evaluated among women and children at risk of undernutrition in LICs because it is possible that differences in the gut microbiome or differences in food matrices may affect how well the markers perform.

In this study, we found that participants could be classified as having consumed the SQ-LNS plus marker compounds within the previous 12 h with high sensitivity and specificity. This window of time would be sufficient for a field-based study, as sample collection typically occurs during daylight hours, which would likely be within 12 h of consumption. Logue et al. (9) have explored the use of urinary saccharin concentrations as a biomarker of recent consumption of saccharin-containing foods and found that urinary concentrations correlated well with dietary intake, and urinary resveratrol metabolite concentrations have previously been used as a biomarker of recent wine consumption with high sensitivity and specificity (12). In our study, the cutoff value we developed for both saccharin and *trans*-resveratrol-3-O-sulfate had 100% specificity. This high specificity would allow researchers of studies or programs with null results to have greater confidence in the findings as it would be unlikely that those who did not adhere to the protocol (nonconsumers) would be misclassified as having adhered to the protocol (consumers). The sensitivity of urinary saccharin and *trans*-resveratrol-3-O-sulfate concentrations as adherence markers was 78% and 89%, respectively. This could lead some consumers to be misclassified as a “nonconsumer,” or as not having adhered to the protocol, and would need to be considered when calculating the power and sample size if those participants were to be excluded from some analyses.

Both saccharin and *trans*-resveratrol were easily incorporated into the SQ-LNS (20 g) at the tested doses. A dose of ∼10 mg saccharin is less than the amount found in 1 packet of Sweet'N Low^®^ (36 mg) manufactured by Cumberland Packing Corporation and is far below the acceptable daily intake (ADI) of 15 mg/kg body weight per day set for saccharin by the US FDA (26) and below the upper bound of the 0–5 mg/kg body weight per day ADI set by the Joint FAO/WHO Expert Committee on Food Additives (27). Similarly, a dose of 5 mg *trans*-resveratrol, which is roughly the amount found in 2 glasses of red wine, is below the US FDA ADI of 0.3 mg/kg body weight per day (28) and a daily dose of 150 mg/d set by the European Food Safety Authority (11). The dosages used in this study would be safe for daily consumption throughout an intervention trial. Acceptability was not formally evaluated in this study, but preliminary research conducted at Nutriset and reports by our study participants did not indicate any adverse organoleptic properties of the SQ-LNS due to the addition of saccharin or *trans*-resveratrol in these amounts. Both compounds appear to be relatively stable in SQ-LNS out to 25 mo post-production when stored at 30°C, but with some loss over time in *trans*-resveratrol at 40°C, which would be sufficient for use in nutritional supplement trials. Additionally, the preliminary results from our small study in children indicate that both saccharin and *trans*-resveratrol may also be useful in study populations in this age group, although this would need to be confirmed in young children in LICs.

Development of an objective marker of adherence is crucial to be able to properly evaluate the impact of an intervention. Abbeddou et al. (1) compared several methods to assess adherence in a study in Burkinabe children and found that adherence based on caregiver report was close to 100% as was tracking through product disappearance rates; however, during 12-h home observation periods, adherence was measured as ∼30%. The low levels of observed adherence coupled with the lack of a change in plasma zinc concentration, which was an intervention outcome, indicates that adherence was likely low in this study and more reliable measurements of adherence are needed. There are several benefits to using saccharin or *trans*-resveratrol as a marker of adherence. First, both compounds are readily available at a reasonable cost for use as food additives, require low dosage for measurement in urine, and are safe for longer-term use at these doses. Second, the results from our child study suggest that it may be possible to evaluate adherence qualitatively using a single spot urine sample instead of collecting urine over 24 h, which is more burdensome for participants. Third, collection of a spot urine sample within 12 h of consumption can be feasible during an in-home or clinic visit, even in nutrition trials in remote and resource-poor settings and in young children when using urine collection bags (29).

### Strengths and limitations

The strengths of the study are that it was well controlled and the portion of porridge containing SQ-LNS was small to ensure that participants consumed the entire sachet of SQ-LNS containing the marker. Additionally, SQ-LNS/S and SQ-LNS/R were added to porridge to simulate a meal that would typically be eaten by the target populations in an LIC. Also, complete urine samples were collected out to 48 h after consumption, which allowed us to determine how long the marker compounds were detectable in the urine.

One limitation of the study design is that complete urine samples were needed. Although the women were instructed on optimal urine collection procedures, 1 woman reported missing all urine from 1 void during a 4-h collection period and 2 others reported missing some urine during a void within a 4-h collection period. These lost samples were very infrequent and therefore unlikely to have an impact on the results. Also, we did not adjust for differences in urine volume between urine samples because this correction was not found to make a difference in either overall excretion patterns or in the sensitivity and specificity of the optimal cutoff points for detecting consumption of SQ-LNS. These findings are consistent with findings from a study measuring urinary resveratrol concentrations (30) and for measurement of other urinary biological indicators (31–34). However, other methods used to adjust for urine volume, such as osmolality or specific gravity, may be appropriate and should be considered in future studies. There was considerable between-person variability in urine concentrations of the target compounds within the 4- or 12-h pooled samples, yet we were still able to correctly classify recent consumption out to 12 h after consumption. Finally, in this study only a single dose of SQ-LNS with target compounds was given, so it is not known how these potential adherence markers would perform after consumption on multiple days or if only partial doses were consumed.

There are also several limitations to the use of these study methods in a field setting. In a community-based study it would be preferable to collect a spot urine sample instead of a 4-h pooled sample, and results from our small study in children indicate this may be feasible. However, this will need to be explored further, as spot urine samples may not perform as well as 4-h pooled samples because of low concentrations of the compounds and/or increased between-person variability in urinary concentrations. We used coolers with cold packs to keep the urine samples cool until they were delivered to the laboratory where boric acid was added as a preservative and the samples were stored at −80°C; however, it may be possible to add a preservative to urine at the time of collection to eliminate the need for refrigeration. Boric acid is not a good candidate for this because it is classified as a reproductive toxin and would not be considered safe for use in a community setting. Alternative preservatives may be acceptable and should be evaluated for this purpose. Another limitation is that the compounds are analyzed by UHPLC-(ESI-)MS/MS, which requires access to sophisticated instrumentation and trained personnel, which may not be available in an LIC. However, it is common for researchers in LICs to collaborate with external laboratories when access to sophisticated instrumentation is not available locally, and a high-throughput method was developed for measuring the compounds in urine to reduce analytical costs (15). Additionally, the participants in our study were asked to refrain from consuming foods and products that contained the compounds for the duration of the study. This would not be feasible in a large community-based study and thus the presence of the compounds in the diet would need to be evaluated first. Dietary intake of the compounds could be assessed using a food-frequency questionnaire and/or by measuring the compounds in urine samples of an intended target population. The markers are intended for use in women of reproductive age and young children in low-income populations in LICs, where the prevalence of micronutrient deficiencies is high and diets are often based on a few dietary staples. Saccharin is not found naturally in any products, but it is used widely in processed foods. Access to saccharin-containing processed foods is likely to vary by population, and saccharin would only be useful as a marker in populations that do not consume these foods regularly. Resveratrol is found in relatively few foods, including red wine and grapes, peanuts, pistachios, and some berries. Except for red wine, which is unlikely to be consumed by the intended target populations, the amount of resveratrol provided by foods is very low and therefore likely to be much lower than the amount of resveratrol (5 mg) that we tested for tracking consumption of SQ-LNS. In the context of a population with background consumption of foods containing resveratrol, a dose of ∼5 mg as an adherence marker would still likely be useful in identifying recent supplement consumption.

### Conclusions

In conclusion, we have shown that urinary concentrations of saccharin and the resveratrol metabolite *trans*-resveratrol-3-O-sulfate reflect recent intake of a single portion of SQ-LNS within 4 h of consumption and can be used to differentiate between consumption and nonconsumption of SQ-LNS for up to 12 h. Further studies are needed to evaluate whether the markers are useful for tracking longer-term consumption of SQ-LNS and whether they can differentiate between high and intermediate and nonconsumption of SQ-LNS. Importantly, although we have demonstrated proof of concept, the markers will need to be evaluated in the intended target populations of women of reproductive age and young children in LICs to determine their usefulness for tracking consumption of SQ-LNS in nutrition interventions in those settings.

## Supplementary Material

nzab089_Supplemental_File

## Notes

Supported by The Bill and Melinda Gates Foundation.

Author disclosures: FBM and MZ are employed by Nutriset S.A.S., the company that developed and manufactured the product used in the study. They had no role in data collection or statistical analysis of results. The other authors report no conflicts of interest.

Supplemental Tables 1–4, Supplemental Methods, and Supplemental Figure 1 are available from the “Supplementary data” link in the online posting of the article and from the same link in the online table of contents at https://academic.oup.com/cdn/.

Abbreviations used: ADI, acceptable daily intake; AUROC, area under the receiver operator curve; LIC, low-income country; ROC, receiver operating characteristic; SQ-LNS, small-quantity lipid-based nutrient supplement(s); SQ-LNS/R, small-quantity lipid-based nutrient supplement containing *trans-*resveratrol; SQ-LNS/S, small-quantity lipid-based nutrient supplement containing sodium saccharin; UC, University of California; UHPLC-(ESI-)MS/MS, ultra-high-performance liquid chromatography interfaced to a mass spectrometer with electrospray ionization.
