# Supplementary material for: Evaluation of Saccharin and Resveratrol as Extrinsic Markers of Small-Quantity Lipid-Based Nutrient Supplement Consumption in Healthy Women
Source: Curr Dev Nutr. 2021 Jul 6;5(7):nzab089. doi: 10.1093/cdn/nzab089 (PMC8302444; doi:10.1093/cdn/nzab089)
Supplement: nzab089_Supplemental_File [file nzab089_supplemental_file.docx]

**Evaluation of saccharin and *trans*-resveratrol as extrinsic markers of small-quantity lipid-based nutrient supplement consumption in healthy women.**

Sarah J. Zyba, Valerie Weinborn, Charles D. Arnold, Arlie L. Lehmkuhler, Fanny B. Morel, Mamane Zeilani, Alyson E. Mitchell, Marjorie J. Haskell

**Online Supplementary Materials**

**Supplementary Table 1.** Nutrient content of a single dose (20g) of small-quantity lipid-based nutrient supplements (SQ-LNS) for pregnant and lactating women.

| Nutrient | SQ – LNS/S | SQ – LNS/R |
| --- | --- | --- |
| Energy | 118 kcal | 118 kcal |
| Proteins | 2.6 g | 2.6 g |
| Lipids | 10 g | 10 g |
| Linoleic Acid | 3.15 g | 3.15 g |
| 𝜶-Linolenic Acid | 0.59 g | 0.59 g |
| Calcium | 280 mg | 280 mg |
| Phosphorus | 190 mg | 190 mg |
| Potassium | 200 mg | 200 mg |
| Magnesium | 65 mg | 65 mg |
| Zinc | 30 mg | 30 mg |
| Copper | 4 mg | 4 mg |
| Iron | 20 mg | 20 mg |
| Manganese | 2.6 mg | 2.6 mg |
| Iodine | 250 𝝁g | 250 𝝁g |
| Selenium | 130 𝝁g | 130 𝝁g |
| Vitamin A | 800 𝝁g | 800 𝝁g |
| Thiamin | 2.8 mg | 2.8 mg |
| Riboflavin | 2.8 mg | 2.8 mg |
| Niacin | 36 mg | 36 mg |
| Pantothenic acid | 7 mg | 7 mg |
| Vitamin B-6 | 3.8 mg | 3.8 mg |
| Folic Acid | 400 𝝁g | 400 𝝁g |
| Vitamin B-12 | 5.2 𝝁g | 5.2 𝝁g |
| Vitamin C | 100 mg | 100 mg |
| Vitamin D | 10 𝝁g | 10 𝝁g |
| Vitamin E | 20 mg | 20 mg |
| Vitamin K | 45 𝝁g | 45 𝝁g |
| ***Sodium saccharin*** | ***10 mg*** | */* |
| ***trans-Resveratrol*** | */* | ***5 mg*** |

**Supplementary Table 2.** Stability of *trans*-resveratrol and sodium saccharin in small-quantity lipid-based nutrient supplement sachets stored at 30º or 40º C at 0, 3, 6, 12, and 25-months post-production^1^.

| Compound | Temperature | T0 | T3 | T6 | T12 | T25 | p-value |
| --- | --- | --- | --- | --- | --- | --- | --- |
| *trans*-Resveratrol | 30ºC | 4.6 ± 0.1 | 3.7 ± 0.6 | 4.4 ± 0.2 | 4.2 ± 0.1 | 5.0 ± 0.0 | 0.004 |
|  | 40º C |  | 4.2 ± 0.1 | 4.0 ± 0.2 | 3.7 ± 0.1 | 3.8 ± 0.1 | <0.001 |
| Sodium saccharin | 30ºC | 9.4 ± 0.5 | 8.7 ± 0.6 | 10 ± 3.0 | 8.7 ± 0.7 | 8.4 ± 2.6 | 0.820 |
|  | 40º C |  | 7.4 ± 0.4 | 8.0 ± 1.4 | 8.8 ± 1.1 | ---- | 0.119 |

^1^T0 = production; T3 = 3 mo post-production; T6 = 6 mo post-production; T12 = 12 mo post-production; T25 = 25 mo post-production. Values are mean ± SD mg; *n* = 3 sachets per time point. Data analyzed using one-way analysis of variance.

**Supplementary Table 3.** Urine concentrations of saccharin and *trans*-resveratrol-3-O-sulfate in healthy women after consuming a single 20 g dose of a small-quantity lipid-based nutrient supplement containing 9.4 mg sodium saccharin or 4.6 mg *trans*-resveratrol^1^.

| **Compound** | **Study day and urine sample** | **Concentration per urine volume (μmol/L urine)** | | **Concentration per g creatinine (μmol/g creatinine)** | |
| --- | --- | --- | --- | --- | --- |
|  |  | **Mean (lower 95% CI, upper 95% CI)** | **Median (Q1, Q3)** | **Mean (lower 95% CI, upper 95% CI)** | **Median (Q1, Q3)** |
| Saccharin | D4U1 | 0.10 (0.10, 0.10) | 0.00 (0.00, 0.00) | 0.10 (0.10, 0.10) | 0.00 (0.00, 0.00) |
|  | D4U2 | 0.10 (0.10, 0.10) | 0.00 (0.00, 0.00) | 0.10 (0.10, 0.10) | 0.00 (0.00, 0.00) |
|  | D4U3 | 0.10 (0.10, 0.10) | 0.00 (0.00, 0.00) | 0.10 (0.10, 0.10) | 0.00 (0.00, 0.00) |
|  | D4U4 | 0.10 (0.10, 0.10) | 0.00 (0.00, 0.00) | 0.10 (0.10, 0.10) | 0.00 (0.00, 0.00) |
|  | D5U1 | 33.7 (17.5, 65.1) | 32.9 (24.6, 74.3) | 92.0 (45.1, 188) | 133 (92.4, 196) |
|  | D5U2 | 9.72 (3.05, 31.0) | 29.3 (13.5, 55.5) | 20.3 (5.44, 75.7) | 80.6 (57.3, 115) |
|  | D5U3 | 3.66 (1.00, 13.32) | 21.3 (0.00, 39.0) | 3.86 (1.03, 14.4) | 22.5 (0.00, 35.5) |
|  | D5U4 | 0.26 (0.10, 0.68) | 0.00 (0.00, 0.00) | 0.25 (0.10, 0.63) | 0.00 (0.00, 0.00) |
|  | D6U1 | 0.10 (0.10, 0.10) | 0.00 (0.00, 0.00) | 0.10 (0.10, 0.10) | 0.00 (0.00, 0.00) |
|  | D6U2 | 0.13 (0.08, 0.21) | 0.00 (0.00, 0.00) | 0.12 (0.08, 0.19) | 0.00 (0.00, 0.00) |
|  | D6U3 | 0.10 (0.10, 0.10) | 0.00 (0.00, 0.00) | 0.10 (0.10, 0.10) | 0.00 (0.00, 0.00) |
|  | D6U4 | 0.13 (0.08, 0.21) | 0.00 (0.00, 0.00) | 0.12 (0.08, 0.19) | 0.00 (0.00, 0.00) |
| *trans*-resveratrol-3-O-sulfate | D4U1 | 0.10 (0.10, 0.10) | 0.00 (0.00, 0.00) | 0.10 (0.10, 0.10) | 0.00 (0.00, 0.00) |
|  | D4U2 | 0.10 (0.10, 0.10) | 0.00 (0.00, 0.00) | 0.10 (0.10, 0.10) | 0.00 (0.00, 0.00) |
|  | D4U3 | 0.10 (0.10, 0.10) | 0.00 (0.00, 0.00) | 0.10 (0.10, 0.10) | 0.00 (0.00, 0.00) |
|  | D4U4 | 0.10 (0.10, 0.10) | 0.00 (0.00, 0.00) | 0.10 (0.10, 0.10) | 0.00 (0.00, 0.00) |
|  | D5U1 | 1.94 (1.13, 3.33) | 2.21 (1.11, 3.19) | 5.37 (2.86, 10.1) | 7.70 (6.18, 9.63) |
|  | D5U2 | 2.10 (1.40, 3.17) | 2.00 (1.46, 2.92) | 5.03 (3.21, 7.89) | 5.29 (4.46, 7.38) |
|  | D5U3 | 1.18 (0.64, 2.18) | 1.11 (0.78, 2.73) | 1.90 (0.93, 3.87) | 3.54 (0.79, 5.45) |
|  | D5U4 | 0.23 (0.13, 0.41) | 0.00 (0.00, 0.74) | 0.24 (0.13, 0.44) | 0.00 (0.00, 0.70) |
|  | D6U1 | 0.18 (0.11, 0.30) | 0.00 (0.00, 0.00) | 0.20 (0.11, 0.36) | 0.00 (0.00, 0.00) |
|  | D6U2 | 0.12 (0.08, 0.16) | 0.00 (0.00, 0.00) | 0.11 (0.09, 0.15) | 0.00 (0.00, 0.00) |
|  | D6U3 | 0.11 (0.09, 0.15) | 0.00 (0.00, 0.00) | 0.11 (0.09, 0.14) | 0.00 (0.00, 0.00) |
|  | D6U4 | 0.10 (0.10, 0.10) | 0.00 (0.00, 0.00) | 0.10 (0.10, 0.10) | 0.00 (0.00, 0.00) |

^1^*n* = 22 women (saccharin) and *n* = 23 women (*trans*-resveratrol-3-O-sulfate). Urine samples collected over 24 hours as three, consecutive 4-hour collection periods (U1-U3) and one, 12-hour overnight collection (U4). Urine sample were collected 24- hours before small-quantity lipid-based nutrient supplement (SQ-LNS) consumption (D4) and for 48- hours after consumption (D5 – D6). SQ-LNS was consumed the morning of D5.

**Supplementary Methods**

*Child study participants.* Four healthy children age 12 – 24 months with mid-upper arm circumference (MUAC) > 114.9 mm, no fever or vomiting within the last 48 hours, no allergy to peanut, cow milk, soy, or saccharin, and who were willing to refrain from consuming dietary sources of the marker compounds for the duration of the study were recruited from a convenience sample from the Davis and the greater San Francisco Bay Area. Written informed consent was provided by at least one parent/ guardian of the child. This study was conducted in the homes of the participants and was approved by the University of California, Davis Institutional Review Board.

*Study procedures:* The study was conducted in October 2020. Due to COVID-19 precautions, all study materials were prepared in advance and dropped off at the participants’ homes before the start of the study. A parent/ guardian measured the child’s MUAC using a provided MUAC tape measure and documented that the child was eligible to participate in the study. Participants received a single dose of small-quantity lipid-based nutrient supplement (SQ-LNS) (10 g) containing 5 mg saccharin and 5 mg *trans*-resveratrol. Participants were provided with a list of foods and products that contained the target compounds and were asked to refrain from consuming anything on the list for the duration of the study. On the day of SQ-LNS consumption, parents/ guardians reported any consumption of the target compounds within the past 3 days or any symptoms of illness within the past 24 hours. Then, a spot urine sample was collected from participants using a pediatric urine collection bag or from a clean child potty if the child was potty trained. The urine was then transferred to a pre-labeled opaque urine container and stored on ice in a cooler until processing. The time of urine collection was documented. Participants were instructed to not consume any foods/ beverages (except water) for at least one hour before SQ-LNS consumption. Participants then consumed the SQ-LNS dose directly from the sachet and were encouraged to consume the whole dose within 30 minutes of the start of the consumption period. Participants were instructed to not consume any foods or beverages (except water) for at least 30 minutes after consuming the SQ-LNS. The sachet weight was measured pre- and post-consumption to estimate the total amount consumed. Thereafter, spot samples from all urine produced over the next ~6 hours were collected using the same method as described above. The next morning parents/ guardians reported any consumption of the target compounds or symptoms of illness within the previous 24 hours and collected a final spot urine sample (roughly 24 hours after SQ-LNS consumption). After all urine samples had been collected, the parents/ guardians placed the cooler with samples on their porch and the study coordinated picked up the cooler and transported it to UC Davis where the samples were immediately processed and stored at -80°C until analysis. Urinary concentrations of the saccharin and resveratrol metabolite *trans*-resveratrol-3-O-sulfate were determined by UHPLC/ESI-MS/MS as described in Weinborn *et al* (1). The urinary concentrations of saccharin and *trans*-resveratrol-3-O-sulfate were plotted by time since SQ-LNS consumption.

**Supplementary Table 4**. Characteristics of study participants, age 12 – 24 months, who completed a study in which they consumed a single serving of small-quantity lipid-based nutrient supplement with 5 mg sodium saccharin and 5 mg *trans*-resveratrol.

| **Participant ID** | **Age (months)** | **MUAC^1^ (mm)** | **Weight (kg)** |
| --- | --- | --- | --- |
| 301 | 22 | 165 | 11.4 |
| 302 | 13 | 165 | 9.3 |
| 304 | 17 | 185 | 13.6 |

^1^Mid-upper arm circumference, MUAC.

**Supplementary Figure 1.** Urinary concentrations of saccharin (A) and *trans*-resveratrol-3-O-sulfate (B) plotted by time (h) since small-quantity lipid-based nutrient supplement (SQ-LNS) consumption from a small (n = 3) proof of concept study in which healthy children age 12 – 24 months consumed 10 g SQ-LNS containing 5 mg saccharin and 5 mg *trans*-resveratrol. Baseline samples reported as 0 h.

**Supplementary References**

1. Weinborn V, Lehmkuhler AL, Zyba SJ, Haskell MJ, Morel FB, Zeilani M, Mitchell AE. Measurement of Saccharin and trans-Resveratrol Metabolites in Urine as Adherence Markers for Small Quantity Lipid-Based Nutrient Supplement Consumption. J Agric Food Chem. 2021;69:1107–14.
